# Effects of Pesticides Commonly Used in Avocado on *Trichogramma exiguum* (Hymenoptera: Trichogrammatidae): A Potential Biological Control Agent for *Stenoma catenifer*

**DOI:** 10.3390/insects17070728

**Published:** 2026-07-15

**Authors:** Michelle Noboa, Ana Barreiro, Jorge Merino, Jorge Espinoza, William Viera, Emely Mora, Wilson Vásquez

**Affiliations:** 1 Programa de Agricultura y Medioambiente, Universidad Santiago de Compostela, 27002 Lugo, Spain; michlle.noboa@iniap.gob.ec (M.N.); ana.barreiro.bujan@usc.es (A.B.); 2 Programa de Fruticultura, Instituto Nacional de Investigaciones Agropecuarias, Quito 170903, Ecuador; jorge.merino@iniap.gob.ec (J.M.); william.viera@iniap.gob.ec (W.V.); 3 Independent Researcher, Riobamba 060501, Ecuador; jorgespinoza5@yahoo.com; 4 Escuela Superior Politécnica de Chimborazo, Sede Orellana, El Coca 220001, Ecuador; 5 Ingeniería Agroindustrial, Universidad de las Américas, Quito 170124, Ecuador; emely.mora@udla.edu.ec

**Keywords:** parasitoid, pesticide selectivity, IOBC, biological control, IPM

## Abstract

*Stenoma catenifer* (Lepidoptera: Depressariidae), the avocado seed borer, represents a major phytosanitary threat in Andean avocado production systems. Egg parasitoids of the genus *Trichogramma* constitute promising biological control agents for this pest; however, their field-level compatibility with agrochemicals commonly used in avocado orchards remains largely uncharacterized. This study evaluated the side effects of 13 commercial pesticide formulations on *Trichogramma exiguum* (Hymenoptera: Trichogrammatidae) under two complementary exposure scenarios: (1) residual contact toxicity on adults and (2) susceptibility of parasitized *Ephestia kuehniella* (Lepidoptera: Pyralidae) eggs, following International Organisation for Biological Control IOBC/WPRS standardized methodology. Corrected adult mortality was assessed at 1 h and 24 h post-exposure, while parasitism expression at the pupal stage, adult emergence rate, and developmental time were recorded for preimaginal stages. At 24 h, abamectin, acephate, lambda-cyhalothrin, and metalaxyl caused 100% adult mortality (harmful), whereas potassium soap and hexythiazox were classified as moderately harmful. In Bioassay 2, potassium soap completely suppressed parasitism expression and adult emergence (0%), while neem extract showed no significant reduction relative to the water control. Developmental time to adult emergence ranged from 9.7 to 12.0 days across treatments, with no statistically significant differences among them. These results indicate that neem extract and the adjuvant dispersant are broadly compatible with *T. exiguum*, whereas neurotoxic insecticides and potassium soap are incompatible under both exposure scenarios. These findings provide an evidence-based compatibility hierarchy to guide the strategic integration of chemical inputs and augmentative biological control in avocado integrated pest management programs.

## Simple Summary

The seed borer *Stenoma catenifer* causes severe economic losses in Andean avocado production. The egg parasitoid *Trichogramma exiguum* represents a promising biological control agent; however, its compatibility with pesticides routinely applied in avocado orchards must be established before field implementation. This study evaluated 13 commercial formulations under two exposure scenarios, residual contact on adults and direct exposure of parasitized host eggs, using IOBC/WPRS standardized protocols. Neem extract demonstrated full compatibility with *T. exiguum*, while neurotoxic insecticides and potassium soap proved categorically incompatible. These findings provide growers with an evidence-based framework for integrating chemical and biological control strategies sustainably.

## 1. Introduction

Avocado (*Persea americana* Mill.) is a crop of increasing global economic importance, yet its market competitiveness is strongly conditioned by pests associated with the fruit and seed [1,2]. Among these, the avocado seed borer *Stenoma catenifer* (Walsingham, 1912) (Lepidoptera: Depressariidae) is recognised as a pest of critical importance in avocado production systems, whose detection in producing areas can activate phytosanitary restrictions and limit fruit trade [1,3]. Larvae of *S. catenifer* penetrate the fruit epidermis and develop internally, feeding on the seed, causing direct losses through internal damage, quality reduction and commercial rejection, as well as complicating early detection due to the cryptic nature of the damage [4].

In lepidopteran pests of agricultural importance, biological control through egg parasitoids constitutes a particularly attractive strategy because it interrupts the pest before larval damage to the fruit occurs. Eggs of *S. catenifer* have been documented to be parasitised by trichogrammatids, supporting the use of egg parasitoids as potential components of IPM programmes [1]. Nava and colleagues reported the selection of *Trichogrammatoidea annulata* and *Trichogramma atopovirilia* for their capacity to parasitise a greater number of *S. catenifer* eggs under laboratory and greenhouse conditions [5], while Hohmann and colleagues underscored the relevance of evaluating egg parasitoids as a key component of IPM schemes given the cryptic nature of the economic damage [6].

The effective adoption of trichogramma in the field depends critically on its compatibility with other management strategies, particularly pesticide applications for concurrent pest or disease management. Comparative bioassays have demonstrated that different pesticides generate differentiated susceptibility responses in *Trichogramma* spp. depending on the active ingredient, mode of action, route and timing of contact, and even the parasitoid species [7]. Laboratory studies have evidenced high vulnerability of trichogramma to broad-spectrum insecticides and considerable variation among chemical classes, with direct implications for the design of chemical-biological integration strategies in agroecosystems [8,9]. Because parasitoids can encounter pesticides through different routes under field conditions, compatibility assessment requires representing at least the following exposure scenarios: (1) residual exposure of adult parasitoids to treated surfaces; and (2) contamination of host eggs after parasitism. Several studies have used IOBC side-effect classification frameworks to categorise substances from ‘innocuous’ to ‘harmful’ for *Trichogramma* spp. [8,10].

The use of *Ephestia kuehniella* Zeller (Lepidoptera: Pyralidae) as a factitious host for rearing and bioassay of *Trichogramma* spp. is a well-established methodological standard in pesticide side-effect testing, endorsed by the IOBC/WPRS Working Group “Pesticides and Beneficial Organisms” [11,12]. Although *E. kuehniella* is not the target host of *Trichogramma* sp., its use as a factitious host is justified on three grounds. Firstly, *E. kuehniella* eggs are the standard substrate used in trichogramma mass-rearing and in all IOBC-compliant side-effect bioassays, ensuring methodological comparability with the existing literature [8,13]. Secondly, the primary objective of the present study was to characterise the intrinsic susceptibility of *T. exiguum* immature stages and adults to pesticide residues, a property determined by the physiology of the parasitoid rather than by the identity of the host egg species; the chorion composition and egg dimensions of *E. kuehniella* are comparable to those of other Lepidopteran hosts used in *Trichogramma* compatibility research [14]. Finally, the quarantine status of *S. catenifer* as a phytosanitary pest in Ecuador precluded its routine maintenance as a laboratory colony under standard conditions. The results of the present study therefore characterise the pesticide susceptibility of *T. exiguum* under controlled laboratory conditions using a validated factitious host system, and constitute the necessary first step in a compatibility assessment framework that should be extended to semi-field conditions with *S. catenifer* as the target host in future research.

The selection of pesticides evaluated in this study was based on actual field-use patterns rather than on a priori assumptions about commonly applied products. Two field workshops were conducted with avocado growers, including exporting producers affiliated with the Corpoaguacate association and smallholder farmers from the Perucho locality (Pichincha, Ecuador). A survey (*n* = 133 respondents) was administered to identify the insecticides, acaricides, and fungicides most frequently applied in avocado orchards. The molecules reported with the highest frequency of use were subsequently selected for inclusion in the bioassays (Table 1), ensuring that the compatibility assessment reflects realistic exposure scenarios for *T. exiguum* under current avocado pest and disease management practices in the study region.

In this context, bioassays were conducted under two complementary exposure scenarios to estimate the risk of pesticides commonly used in avocado orchards to *T. exiguum*, and to provide technical criteria for integrating chemical and biological strategies without compromising the effectiveness of biological control or the sustainability of the crop management system in Andean production regions of Ecuador.

## 2. Materials and Methods

### 2.1. Study Site and Environmental Conditions

Experiments were conducted at the research laboratory of Universidad de las Américas, located at 0°9′47.7″ S, 78°27′31.3″ W, approximately 2850 m a.s.l. (Quito, Ecuador). All bioassays were performed in a climate-controlled chamber at 26 ± 2 °C, 60 ± 8% RH and a 12:12 h L:D photoperiod.

### 2.2. Insect Colonies

#### 2.2.1. Rearing of *Ephestia kuehniella*

Rearing began with larvae collected from almond flour and placed (≥50 individuals) into plastic boxes (26 × 26 × 15 cm) fitted with tulle fabric for ventilation. Each box received 250 g of ground flaxseed for food and insects were kept there for approximately 16 days until adult emergence. Newly emerged adults were transferred in groups of 25 individuals to transparent plastic cylinders closed at both ends with tulle fabric. Ovipositions were collected daily. Prior to use in bioassays, *E. kuehniella* eggs were killed by UV irradiation (30 min) to prevent hatching while preserving chorion integrity [12].

#### 2.2.2. Rearing of *Trichogramma exiguum*

Parasitized eggs of a host belonging to the superfamily Noctuoidea were collected on avocado leaves in San José de Minas, Pichincha, Ecuador (0°09′52.6″ N, 78°25′09.8″ W). The emerging parasitoids were identified as *Trichogramma exiguum* through both classical taxonomy and molecular techniques and were subsequently used to establish the experimental population. Eggs of *Ephestia kuehniella* were UV-irradiated [15] and were mounted on cardboard strips (0.5 cm wide × 8.0 cm long) using a 50% (*v*/*v*) gum arabic solution. Each strip was placed inside a glass test tube (10 mm in diameter × 100 mm in length) and exposed to the *T. exiguum* population for parasitization. A drop of honey was provided on the inner wall of each tube as a food source for adult parasitoids. The tubes were maintained at 26 ± 2 °C, 70 ± 8% relative humidity and a 12:12 h (L) photoperiod for approximately 10 days, until the emergence of the subsequent generation of *T. exiguum* adults.

### 2.3. Pesticides

Based on the field survey described in the i ntroduction, fourteen treatments were evaluated; these comprised potassium soap along with 13 commercial pesticide formulations; these comprised potassium soap along with 13 commercial pesticide formulations—including insecticides (lambda-cyhalothrin [IRAC 3A], abamectin [IRAC 6], acephate [IRAC 1B]), an acaricide (hexythiazox [IRAC 10A]), fungicides (metalaxyl [FRAC 4], thiophanate [FRAC 1], azoxystrobin [FRAC 11], thiabendazole [FRAC 1]), biopesticides and botanical extracts (paraffinic oil, neem extract, argemone extract) and an adjuvant (dispersant)—and a water control (Table 1). For each product, the working concentration corresponded to the maximum dose recommended by the manufacturer for field application. Solutions were prepared with distilled water to a final volume of 500 mL and transferred to labelled spray bottles.

### 2.4. Bioassay 1: Residual Contact Toxicity on Adult T. exiguum

**Figure 1:**
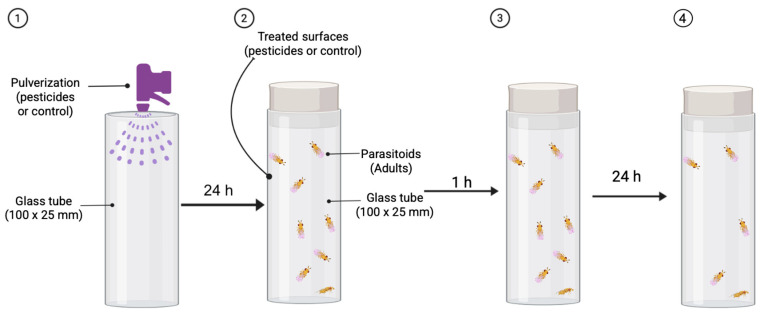
Bioassay for contact toxicity. (1) Spraying into glass tubes and releasing adults of *Trichogramma exiguum*; (2) Introduction of adults 24 h after pesticide application; (3) Evaluation 1 h after contact with the treated surface; (4) Evaluation 24 h after contact with the treated surface. 26 ± 2 °C, 70 ± 8% relative humidity and a 12:12 h (L) photoperiod.

To assess residual toxicity, inner surfaces of glass vials (100 mm length × 25 mm diameter) were uniformly sprayed with each of the 14 solutions, solid-formulated pesticides were weighed on an analytical balance (Mettler Toledo, Greifensee, Switzerland), and the amount corresponding to the maximum recommended field dose was calculated and dissolved in 100 mL of distilled water. Liquid-formulated pesticides were measured using adjustable micropipettes (Thermo Scientific, MA, USA; 10–100 μL), and the volume corresponding to the maximum recommended field dose was calculated and diluted in 100 mL of distilled water, using a manual sprayer that produced droplets of 80–100 µm and atomized approximately 500 ± 20 µL of the solution onto the inner surface of each vial (Figure 1). Treated vials were left undisturbed for 24 h at room temperature to allow complete evaporation of the distilled water carrier. Each glass vial constituted the experimental unit, and five replicate vials were used per treatment. An average of 20 (12–47) adult *T. exiguum* individuals were introduced into each replicate vial and sealed with Parafilm^®^. Adult mortality was assessed at 1 h and 24 h after introduction. Mortality was confirmed by gentle stimulation with a single-bristle brush; insects in a supine position and completely immobile were recorded as dead. Corrected mortality (%) was calculated using Abbott’s formula [16]:

$$Corrected\;mortality\;\left(\%\right)=\left[\frac{\left(T\;-\;C\right)}{\left(100\;-\;C\right)}\right]\;\times \;100$$

where *T* is observed mortality in the treatment and *C* is natural mortality in the distilled water control.

Treatments were classified according to IOBC hazard categories: Category 1 = innocuous (<30% mortality); Category 2 = slightly harmful (30–79% mortality); Category 3 = moderately harmful (80–99% mortality); and Category 4 = harmful (>99% mortality) [11].

### 2.5. Bioassay 2: Susceptibility of Parasitised Eggs

The bioassay assessing the susceptibility of parasitized eggs followed the standard IOBC/WPRS methodology for testing the effects of pesticides on egg parasitoids [11,12], specifically while they were developing within the host egg. Each cardboard card constituted the experimental unit, and five replicate cards were used per treatment. Batches of approximately 30 (21–36) UV-irradiated eggs of *E. kuehniella* were mounted on each replicate card (light blue cardboard, 2 × 2 cm), and each card was individually placed in a glass vial (10 mm diameter × 100 mm length) containing approximately 15–20 adult females of *T. exiguum* (24–48 h old) and a small drop of honey as a food source. Eggs and parasitoid adults were kept for 48 h at 26 ± 2 °C, 70 ± 8% relative humidity, and a 12:12 h photoperiod to allow parasitism to occur.

**Figure 2:**
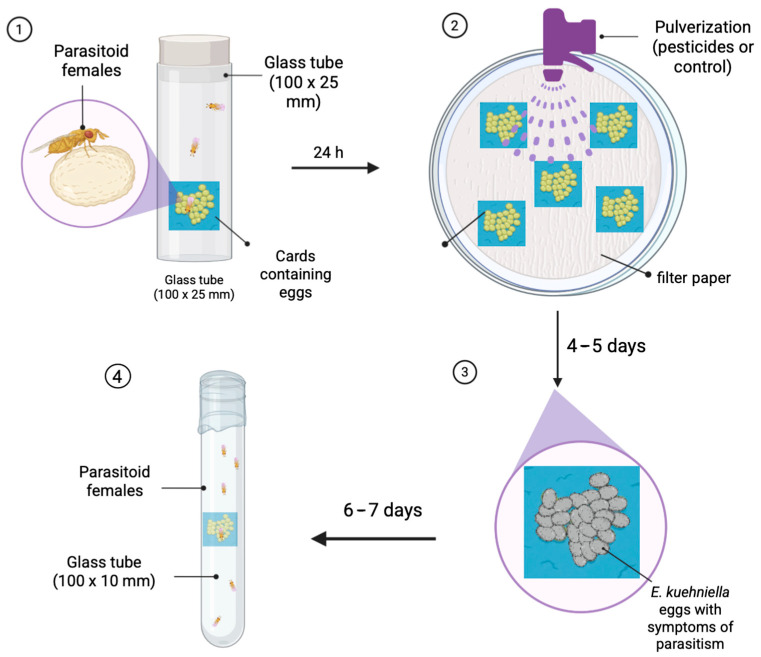
Bioassay for susceptibility. (1) Exposure of the cards containing *Ephestia kuehniella* eggs to parasitism by *Trichogramma exiguum* females; (2) Spraying the cards post-parasitism with pesticides or the control; (3) Evaluation of the pupal development of *T. exiguum* in egg colouration; (4) Evaluation of the effects on adult emergence. 26 ± 2 °C, 70 ± 8% relative humidity and a 12:12 h (L) photoperiod.

At the end of the 48 h exposure period, adult parasitoids were removed and pesticides were applied at the concentrations indicated in Table 1; solid-formulated pesticides were weighed on an analytical balance (Mettler Toledo, Switzerland), and the amount corresponding to the maximum recommended field dose was calculated and dissolved in 100 mL of distilled water. Liquid-formulated pesticides were measured using adjustable micropipettes (Thermo Scientific, USA; 10–100 μL), and the volume corresponding to the maximum recommended field dose was calculated and diluted in 100 mL of distilled water, using a manual sprayer that produced droplets of 80–100 µm and atomized approximately 500 ± 20 µL of the solution over each card (Figure 2). Each card was then allowed to air-dry for 24 h before being transferred again to sterile glass vials (Figure 2). Development of *T. exiguum* at the pupal stage was evaluated by counting the proportion of eggs per card that had developed the characteristic metallic gray coloration resulting from embryogenesis of the genus *Trichogramma*. The mean parasitism rate, determined based on the distilled water control replicates, was 83.9 ± 14.8% eggs per card. This is consistent with parasitism rates reported for *T. exiguum* on *E. kuehniella* under comparable laboratory conditions [17]. Vials were inspected periodically until adult emergence ceased.

Three response variables were recorded for each egg card:

1. Parasitism expression at the pupal stage (%):

$$PEPS\;\left(\%\right)=\frac{Number\;of\;black\;eggs}{Total\;number\;of\;eggs\;per\;card}\times 100\;$$

2. Adult emergence (%):

$$AE\;\left(\%\right)=\frac{Number\;of\;emerged\;adults}{Total\;number\;of\;eggs\;per\;card}\times 100$$

3. Time to adult emergence (days) was recorded from pesticide application to first adult emergence.

For both parasitism expression at the pupal stage (%) and adult emergence (%), values were corrected using Abbott’s formula relative to the water control treatment. This correction was applied to account for the inherent variability associated with biological processes and to provide a more accurate estimate of the effects attributable to pesticide exposure. Corrected values were calculated as:

$$Corrected\;\left(\%\right)\;=\;1\;-\;\left(\frac{nT}{nC}\right)\;\times \;100\;$$

where *nT* is the number of individuals recorded in the pesticide-treated group and *nC* is the number of individuals recorded in the water control [16]. The use of Abbott’s correction allowed the pesticide-induced effects on the preimaginal stages of *T. exiguum* developing within the host egg to be quantified independently of natural variation observed under control conditions.

### 2.6. Statistical Analysis

#### 2.6.1. Bioassay 1

Corrected adult mortality (%) was analysed separately at 1 and 24 h. Model assumptions were verified using Shapiro–Wilk and Levene’s tests; proportion data were arcsine square-root transformed where needed. A one-way analysis of variance (ANOVA) followed by Tukey’s honestly significant difference (HSD; α = 0.05) was applied when assumptions were satisfied; when assumptions could not be met after transformation, the Kruskal–Wallis test followed by Dunn’s test with false discovery rate (FDR) correction was used.

#### 2.6.2. Bioassay 2

Parasitism rate and adult emergence percentage data were subjected to a one-way analysis of variance (ANOVA), with pesticide as the treatment factor. Since both variables represent proportions or percentages, prior transformations were applied to stabilize variance and approximate the normality of the residual distribution: the parasitism rate was transformed using the function $\sqrt{\left(x\;+\;0.5\right)}$, while the adult emergence percentage was transformed using the arcsine square root transformation arcsin $\left[\sqrt{\left(\frac{x}{100}\right)}\right]$. Descriptive statistics (mean, standard deviation, standard error, minimum, and maximum) were calculated and reported on the original scale. When the ANOVA *F*-value was statistically significant (*p* < 0.05), mean separation was performed using the Scott–Knott test at the same significance level [18]. Model assumption validity was assessed using the Shapiro–Wilk test for residual normality and Bartlett’s test for homogeneity of variances (Table S1). All analyses were conducted using RStudio software version 2026.01 [19].

### 2.7. Methodological Limitations

A key methodological consideration is that, although topical and/or residual application is the standard method prescribed by IOBC laboratory testing protocols, it does not reproduce the nature or intensity of pesticide exposure that *T. exiguum* would experience under field conditions, since the maximum field-recommended dose was used for each compound. Consequently, the mortality rates obtained under topical exposure are expected to overestimate the risk posed under field conditions. These results should therefore be interpreted as worst-case ecotoxicological indicators rather than as direct predictors of population-level impacts in commercial orchards.

An additional methodological limitation was the inability to introduce an exact number of individuals per vial, owing to the handling constraints imposed by the small body size of *T. exiguum*. Precise counting and transfer of adults inevitably increases physical manipulation, which may render individuals susceptible to injury or mortality prior to the onset of pesticide exposure, potentially introducing uncontrolled variation into the mortality estimates.

Additionally, in Bioassay 2, the factitious host *Ephestia kuehniella* was used owing to its ease of laboratory rearing, under the assumption that its egg characteristics would behave similarly to those of the lepidopteran target host, *Stenoma catenifer*. Nevertheless, we consider that field or semi-field validation studies, incorporating realistic exposure scenarios and population-level parameters, are warranted to confirm the practical compatibility of the evaluated products with *T. exiguum* under avocado agroecosystem conditions.

## 3. Results

### 3.1. Bioassay 1: Residual Contact Toxicity on Adult T. exiguum

Model assumption checks indicated that corrected adult mortality did not meet the requirements for ANOVA. At 24 h, Levene’s test revealed significant heterogeneity of variances (*F* = 3.15, *p* = 0.0014), and several treatments (distilled water control, abamectin, lambda-cyhalothrin, acephate, and metalaxyl) showed zero variance due to 100% mortality across all replicates. At 1 h, although variances were homogeneous (Levene, F = 1.24, *p* = 0.28), the distilled water control similarly exhibited zero variance, violating the assumptions required for parametric analysis. Accordingly, the Kruskal–Wallis test was applied at both evaluation times, and corrected mortality differed significantly among pesticide treatments, with mean separation performed using Dunn’s test with FDR correction.

At 1 h post-application, mortality was generally low across treatments, ranging from 0% (water control) to 37.5 ± 7.11% (acephate). Dunn’s test identified acephate as the only treatment causing significantly higher mortality than the dispersant and water control, which shared the lowest values. All remaining pesticides were statistically similar to each other. Based on the IOBC classification, acephate and lambda-cyhalothrin were rated as slightly harmful (Category 2), while all other treatments were considered innocuous (Category 1) at this time point (Table 2).

At 24 h post-application, mortality increased markedly for most treatments. Abamectin, acephate, lambda-cyhalothrin, and metalaxyl caused complete mortality (100 ± 0.00%; letter a), and were classified as harmful (IOBC category 4). Potassium soap (95.1 ± 2.16%) and hexythiazox (88.4 ± 6.80%) produced similarly high mortality and were rated as moderately harmful (Category 3). Thiabendazole, neem extract, paraffinic oil, azoxystrobin, berberine + argemone, and thiophanate caused intermediate mortality levels ranging from 44.3 to 73.2% and were classified as slightly harmful (Category 2). The dispersant (14.3 ± 1.92%) and water control (0 ± 0%) caused negligible mortality and remained innocuous (Category 1).

**Figure 3:**
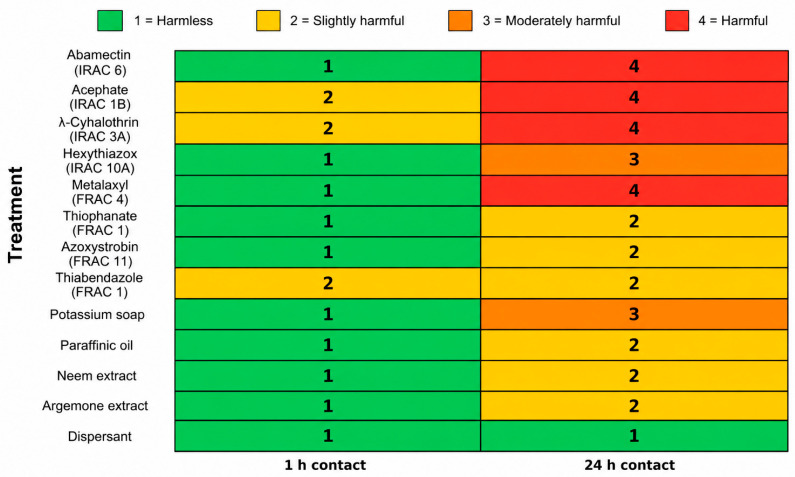
Heatmap showing the harmfulness profile of pesticides to adult *Trichogramma exiguum* at 1 h and 24 h after residual exposure (bioassay 1). IOBC harmfulness categories were defined as follows: 1 = harmless, 2 = slightly harmful, 3 = moderately harmful, and 4 = harmful.

None of the pesticides evaluated proved completely innocuous at 24 h post-application (Figure 3). Nevertheless, products such as azoxystrobin, berberine + argemone, and thiophanate, which caused corrected adult mortality ranging from 44 to 63%, could be considered for strategic application windows, for instance, during periods when parasitoids are not actively foraging in the field, as part of an integrated pest management approach aimed at preserving natural enemy populations while maintaining adequate crop protection.

### 3.2. Bioassay 2: Susceptibility of Parasitised Eggs

#### 3.2.1. Parasitism Rate in Pupae Expression of *T. exiguum*

The parasitism rate differed significantly among pesticide treatments (ANOVA, *p* = 0.00178). Mean separation using the Scott –Knott test identified three distinct groups (Table 3). The results for parasitism expression reveal a compatibility spectrum between the evaluated pesticides and *Trichogramma exiguum*, with direct implications for integrated pest management. Potassium soap showed absolute incompatibility with the parasitoid, completely suppressing parasitism expression (0%); therefore, its application should be carried out prior to parasitoid releases. Lambda-cyhalothrin (22.0%) and thiabendazole (9.45%) were placed at an intermediate-to-high risk level, as their drastic reductions relative to the control suggest severe lethal or sublethal effects on adults or on the parasitoid’s intraovarial development. Paraffinic oil (31.05%), abamectin (32.0%), argemone + berberine (40.89%), acephate (43.3%), hexythiazox (49.7%) and metalaxyl (49.7%), although statistically different from the control, retained moderate parasitism rates that could be considered compatible if re-entry intervals or pre-release withholding periods are properly managed. Finally, neem extract (82.33%), showing no significant difference from the untreated control, represents the most compatible alternative with parasitoid activity, making its inclusion in integrated management strategies feasible without compromising the efficacy of *T. exiguum* as a biological control agent.

#### 3.2.2. Adult Emergence

Adult emergence differed significantly among pesticide treatments (ANOVA, *p* = 0.000146), revealing a clear gradient of toxicity toward *Trichogramma exiguum*. The water control and neem extract yielded the highest emergence rates (96.0 ± 2.4% and 82.33 ± 7.5%, respectively), with no significant difference between them, confirming that neem does not compromise the completion of the parasitoid’s life cycle and is therefore fully compatible with augmentative biological control programs. All remaining treatments produced significantly lower emergence rates: metalaxyl (49.7 ± 2.1%), acephate (43.3 ± 8.6%), hexythiazox (44.0 ± 8.3%), argemone + berberine (40.89 ± 2.3%), paraffinic oil (31.05 ± 6.6%), abamectin (26.0 ± 1.1%), lambda-cyhalothrin (14.7 ± 8.4%), and thiabendazole (9.45 ± 5.9%), collectively forming an intermediate group with moderate-to-severe impacts on adult production. Potassium soap completely suppressed adult emergence (0 ± 0.0%), representing the most harmful treatment for this variable and rendering it entirely incompatible with any strategy involving *T. exiguum* releases.

Particularly is noteworthy the case of abamectin and lambda-cyhalothrin, for which adult emergence rates (26.0% and 14.7%, respectively) were markedly lower than their corresponding parasitism expression values (32.0% and 22.0%), indicating that these compounds exert a dual detrimental effect: they not only reduce the initial parasitism rate but also impair larval or pupal development within the host egg, preventing the successful completion of the parasitoid’s life cycle even in eggs that had already been parasitized.

#### 3.2.3. Time to Emergence

Development time from egg to adult ranged from 9.7 to 12.0 days across treatments, representing a relatively narrow window of variation. None of the treatments produced development times that deviated markedly from the water control (11.0 ± 0.0 days), suggesting the absence of biologically meaningful differences among them. The shortest development times were recorded under paraffinic oil (9.7 ± 0.6 days) and acephate (10.0 ± 0.0 days), while metalaxyl and thiabendazole produced the longest durations (12.0 ± 0.7 and 12.0 ± 0.4 days, respectively), Hexythiazox (11.0 ± 0.0 days), argemone + berberine (11.2 ± 0.5 days), λ-cyhalothrin (11.3 ± 0.2 days), neem (11.4 ± 0.5 days), and abamectin (11.7 ± 0.6 days) all remained closely aligned with the control value. Potassium soap was excluded from this analysis, as parasitized host eggs never reached eclosion under this treatment, an outcome attributable to desiccation caused by the surfactant properties of the soap, which likely disrupted the integrity of the egg chorion and prevented the completion of embryonic development.

## 4. Discussion

### 4.1. Residual Contact Toxicity on Adult T. exiguum (Bioassay 1)

The 100% corrected adult mortality recorded for acephate, lambda-cyhalothrin, abamectin, and metalaxyl after 24 h of contact, with IOBC category 4 classification, is consistent with the documented toxicity mechanisms of these chemical groups on non-target insects. Acephate is a systemic organophosphate whose toxicity is exerted primarily through its active metabolite, methamidophos, which irreversibly inhibits acetylcholinesterase (AChE) at nerve synapses, causing acetylcholine accumulation and overstimulation of nicotinic and muscarinic receptors [20,21]. The temporal dynamics observed, moderate mortality at 1 h and total mortality at 24 h, are congruent with the time required for biotransformation of acephate to its active metabolite within the parasitoid organism [22]. Similar results have been reported by Pazini and colleagues [23] for the parasitoid *Telenomus podisi* (Hymenoptera: Platygastridae), confirming that organophosphates compromise synaptic transmission in parasitoids in a manner equivalent to target pests.

The toxicity of lambda-cyhalothrin, a type II pyrethroid, operates through prolonged binding to voltage-dependent sodium channels in neuronal membranes, generating persistent depolarisation, hyperexcitability and death [24]. The moderate mortality observed at 1 h (31.5 ± 11.85%) and total mortality at 24 h underscores the importance of including delayed evaluation times in selectivity bioassays, as immediate assessments may substantially underestimate residual risk. Similarly lambda-cyhalothrin was found to be among the most harmful compounds for *T. podisi* in irrigated rice, confirming its incompatibility with biological pest control strategies [25].

All pesticides exhibited a marked increase in corrected adult mortality between the 1 h and 24 h evaluation periods (Figure 3). This pattern suggests that short-term assessments substantially underestimate the actual hazard posed to adult parasitoids, as the lethal effect appears not to be immediate but rather dependent on exposure duration or cuticular absorption over time. Consequently, relying solely on early-time evaluations may lead to inaccurate safety classifications with relevant implications for biological control programs. Furthermore, insecticides and acaricides with neurotoxic modes of action were the compounds responsible for 100% mortality at 24 h, confirming their incompatibility with biological control strategies involving adult parasitoids and underscoring the need to exclude them from integrated pest management programs where *Trichogramma* spp. or related natural enemies are deployed.

The 100% mortality induced by abamectin at 24 h is particularly noteworthy, as this compound is frequently described as having a low impact on natural enemies. Abamectin is an avermectin that acts as an agonist of GABA-gated chloride channels, causing irreversible neuronal hyperpolarisation and death [26]. The high sensitivity of *T. exiguum* in the glass vial bioassay may reflect species-specific differences in cytochrome P450-dependent detoxification capacity and/or the greater relative contact surface of the glass substrate compared to plant surfaces. As Desneux and colleagues [26] noted, mortality in acute toxicity bioassays may represent only a partial measure of the real impact of pesticides on beneficial arthropods.

Regarding the 100% mortality induced by metalaxyl at 24 h (IOBC category 4), this constitutes one of the most unexpected findings of the present study. Metalaxyl is a phenylamide fungicide whose primary target in oomycetes is the RNA polymerase I complex of the rRNA gene transcription machinery [27], a target with no recognised equivalent in insect physiology. However, the non-target toxicity of metalaxyl on beneficial arthropods has been reported previously [26]. Several possible routes may account for the observed toxicity. Firstly, metalaxyl and its active enantiomer mefenoxam possess considerable lipophilicity (log K_ow_ ≈ 1.65), which may facilitate passive diffusion across the arthropod cuticle and non-specific disruption of membrane-bound enzymatic systems [28]. Secondly, phenylamide fungicides have been reported to inhibit cytochrome P450 monooxygenases in non-target organisms, potentially compromising oxidative detoxification capacity and rendering parasitoids more vulnerable to co-formulant constituents [29]. Thirdly, the commercial formulation evaluated (PREVIL^®^) contains both metalaxyl and propamocarb; the latter is a carbamate-type fungicide whose inhibition of phospholipid biosynthesis in oomycetes may produce secondary surfactant-like effects on arthropod cuticle integrity at field-use concentrations [30]. This contrasts markedly with Pazini and colleagues [25], who found that most fungicides are innocuous (Category 1) for *T. podisi*, underscoring the need for species-specific evaluations even for fungicide compounds before assuming compatibility with biological control.

Two other products classified as IOBC category 3, hexythiazox (88.4 ± 6.80% at 24 h) and potassium soap (95.1 ± 2.16%), warrant special consideration, as both are frequently perceived as low-risk inputs [31,32]. The selective acaricidal action of hexythiazox on immature mite stages does not predict its impact on parasitoid Hymenoptera; its lipophilic structure may facilitate cuticular penetration and induce subacute toxicity through alternative metabolic pathways [26]. For potassium soap, the observed progressive mortality, low at 1 h (7.72 ± 2.80%) and sharply elevated at 24 h, is consistent with a cumulative desiccation mechanism rather than acute neurotoxicity. The partial rehydration of soap residues at 70% RH, combined with the frequent antennal contact behaviour of *Trichogramma* spp. during host-seeking [33], may have amplified cuticular disruption in the sealed glass vial environment. This contrasts with the expected low residual activity of potassium soap on plant surfaces under field conditions [34], emphasising the need for caution in extrapolating glass vial results directly to field compatibility assessments [26].

The botanical compounds and fungicides—including neem extract (69.9%), azoxystrobin (62.6%) and thiophanate (44.3%)—produced intermediate mortalities at 24 h (Category 2: 44–73%). The residual contact toxicity of neem extract may reflect azadirachtin’s multi-site mode of action, which includes endocrine disruption and direct cytotoxic effects on insect tissues [35]. As demonstrated by Turchen and colleagues [36], the absence of acute mortality does not guarantee compatibility with biological control, since azadirachtin can significantly compromise parasitism and progeny production in exposed generations. The dispersant (14.3 ± 1.92%; Category 1) was the only compound classified as innocuous at 24 h, confirming its suitability for co-use with *T. exiguum* under residual contact conditions.

The progressive toxicity observed between 1 and 24 h demonstrates that exposure time is a critical determinant of residual toxicity for several compounds, particularly those with delayed biotransformation pathways (acephate and abamectin) or physical desiccation mechanisms (potassium soap). This reinforces the methodological recommendation that IOBC bioassays should always include multiple temporal evaluations, particularly 24 h post-exposure assessments, to capture the full toxicological profile of pesticide residues on parasitoid Hymenoptera.

### 4.2. Susceptibility of Parasitised Eggs

The wide variation in parasitism rates and adult emergence observed among the evaluated treatments reflects a toxicity spectrum ranging from complete incompatibility to full compatibility, consistent with the patterns described by Smith (1996) [37] for other *Trichogramma* species exposed to different pesticide groups. This gradient highlights the importance of considering multiple biological parameters, rather than relying solely on adult mortality, when assessing the selectivity of pesticides within augmentative biological control programs.

Potassium soap proved completely incompatible with *Trichogramma exiguum*, causing a 100% reduction in both parasitism and adult emergence. This finding is consistent with the mode of action of potassium salts of fatty acids, which act through direct contact by penetrating the insect cuticle, leading to dehydration and disruption of cellular membranes [38]. Haddi and colleagues [39] reported that insecticidal soaps may exert both direct and indirect harmful effects on natural enemies, including predators and parasitoids, thereby compromising biological control programs. Consequently, the most appropriate management strategy would be to apply potassium soap sufficiently in advance of *T. exiguum* releases, allowing residues to dissipate before parasitoid establishment in the field.

The case of abamectin, which resulted in 32.0% parasitism and 26.0% adult emergence, is consistent with the findings of Hassan and colleagues [40], who reported a similar pattern in selectivity studies involving different *Trichogramma* species. These authors emphasized that reduced adult emergence relative to the initial parasitism rate provides direct evidence of toxicity affecting immature developmental stages. Specifically for abamectin, Manzar and colleagues [41] demonstrated that treatments applied to eggs parasitized by *Trichogramma chilonis* resulted in emergence rates below 33.9%, with even lower values observed when exposure occurred during larval or pupal stages.

These findings highlight the ability of abamectin to penetrate the egg chorion and adversely affect the internal development of the parasitoid. Abamectin is a highly lipophilic avermectin whose transovarial penetration has been documented in several arthropod species, and this physicochemical property may explain the lethal effects observed on *T. exiguum* larvae and pupae developing within the host egg. In this context, Desneux and colleagues (2007) [26] cautioned that the sublethal effects of pesticides on parasitoids are often underestimated when assessments are restricted to adult mortality, as preimaginal development and effective parasitism constitute more sensitive indicators of pesticide compatibility.

The absence of treatment-induced alterations in developmental rate among the remaining pesticides suggests that these compounds primarily exert their detrimental effects on early-stage processes, such as host acceptance, oviposition behavior, or egg viability, rather than on embryonic or larval development per se, implying that sublethal exposure does not appear to disrupt the physiological machinery governing development speed in individuals that successfully complete their life cycle.

From an applied perspective, this consistency is particularly relevant for field implementation: even when moderately compatible pesticides are used, adult emergence remains predictable within an approximately 10–12 day window, thereby facilitating the synchronization of *T. exiguum* releases with pest egg availability and preserving the operational reliability of biological control programs.

## 5. Conclusions

The results of the present study on adult *T. exiguum* exposed to pesticide residues (Bioassay 1) are largely consistent with the findings obtained for preimaginal stages developing within parasitized host eggs (Bioassay 2), though notable divergences between the two exposure scenarios provide critical additional information for IPM decision-making. Neem extract and the dispersant were the only compounds that demonstrated acceptable compatibility across both bioassays, indicating a strong likelihood of compatibility of these inputs with augmentative releases of *T. exiguum* in avocado pest management programs.

Acephate, lambda-cyhalothrin, abamectin and metalaxyl proved categorically incompatible with *T. exiguum*, causing 100% adult mortality at 24 h (IOBC Category 4) and significant reductions in parasitism expression and adult emergence, confirming their incompatibility across both exposure routes and underscoring the need to exclude them from any spray program concurrent with parasitoid releases. Potassium soap constituted a special case of absolute incompatibility in Bioassay 2; unlike neurotoxic insecticides, however, its physical mode of action and rapid environmental degradation suggest that temporal segregation from parasitoid releases may represent a viable strategy. None of the fungicides evaluated demonstrated complete innocuousness across both bioassays, a finding that contrasts with the general assumption that fungicides pose negligible risk to parasitoid Hymenoptera, and one that calls for greater caution in the a priori classification of this chemical group as compatible with biological control.

These findings provide growers and extension services with an evidence-based compatibility hierarchy that can guide the strategic integration of chemical inputs and augmentative biological control, contributing to more sustainable crop protection in avocado production systems. Future research should extend these conclusions to greenhouse and field conditions using *S. catenifer* as the target host, and should incorporate sublethal and transgenerational endpoints to capture the full spectrum of pesticide effects on *T. exiguum* population dynamics.

## Figures and Tables

**Table 1:** Identity, dosage and mode of action of biorational pesticides commonly used in avocado orchards and treatments evaluated on *Trichogramma exiguum*.

No	Active Ingredient	Dose * (µL/100 mL)	Trade Name	Formulation	Chemical Group	Mode of Action	IRAC/FRAC Classification	AI Concentration
1	Lambda-cyhalothrin	150	XANDA^®^	Liquid	Pyrethroids	Sodium channel modulator	IRAC 3A	141 g/L
2	Abamectin	40	NEW MECTIN^®^	Liquid	Avermectins	Chloride channel activator	IRAC 6	18 g/L
3	Acephate	0.1 **	TROFEO^®^	Soluble powder	Organophosphates	Acetylcholinesterase inhibitor	IRAC 1B	75% *w*/*w*
4	Hexythiazox	0.05 **	ESTRUENDO^®^	Soluble powder	Thiazolidinediones	Growth inhibitor	IRAC 10A	100 g/kg
5	Metalaxyl	170	PREVIL^®^	Liquid	Metalaxyl + Propamocarb	RNA synthesis inhibitor	FRAC 4	15% + 10%
6	Thiophanate	150	NOVAK^®^	Liquid	Benzimidazoles	Mitosis inhibitor	FRAC 1	50% *w*/*v*
7	Azoxystrobin	250	AMISTAR TOP^®^	Liquid	Strobilurins	Mitochondrial respiration inhibitor	FRAC 11	20% *w*/*v*
8	Thiabendazole	100	MERTECT^®^	Liquid	Benzimidazoles	Microtubule assembly inhibitor	FRAC 1	42.9% *w*/*w*
9	Potassium soap	250	KLEAN-K^®^	Liquid	Surfactants	Lipid membrane disruptor, contact action	Not classified (IRAC)	22% *w*/*v*
10	Paraffinic oil	100	AGRICOL^®^	Liquid	Paraffins	Physical action by suffocation/contact	Not classified (IRAC)	78.37% *w*/*w*
11	Azadirachtin (Neem extract)	500	NEEM-X^®^	Liquid	Tetranortriterpenoid limonoid	Feeding and growth inhibitor	Not classified (IRAC)	4 g/L
12	Chelerythrine and Berberine (Argemone extract)	250	PRONTIUS IO^®^	Liquid	Benzylisoquinoline alkaloid	Insecticidal activity	Not classified (IRAC)	179.76 g/L
13	Dispersant	50	AGRAL^®^	Liquid	Surfactants	Adjuvant—no direct insecticidal action	Not classified (IRAC)	200 g/L
14	Distilled water (control) ***	-	-	Distilled water	-	-	-	-

* Recommended dose by the manufacturer under field conditions, converted to µL per 100 mL. ** g/100 mL. IRAC = Insecticide Resistance Action Committee; FRAC = Fungicide Resistance Action Committee; RNA = ribonucleic acid. *** Distilled water volume 100 mL.

**Table 2:** Corrected adult mortality (%) of *Trichogramma exiguum* at 1 and 24 h post-introduction into glass vials treated with pesticide residues (mean ± SE).

Pesticide	Corrected Mortality (%)			
	1 h Post Application	IOBC Category 1 h	24 h Post Application	IOBC Category 24 h
Abamectin	21.4 ± 6.80 ab	1. Innocuous	100 ± 0.00 a	4. Harmful
Acephate	37.5 ± 7.11 a	2. Slightly harmful	100 ± 0.00 a	4. Harmful
λ-cyhalothrin	31.5 ± 11.85 ab	2. Slightly harmful	100 ± 0.00 a	4. Harmful
Metalaxyl	15.3 ± 3.67 ab	1. Innocuous	100 ± 0.00 a	4. Harmful
Potassium soap	7.72 ± 2.80 ab	1. Innocuous	95.1 ± 2.16 ab	3. Moderately harmful
Hexythiazox	19.7 ± 6.89 ab	1. Innocuous	88.4 ± 6.80 abc	3. Moderately harmful
Thiabendazole	29.6 ± 10.33 ab	1. Innocuous	73.2 ± 3.47 abc	2. Slightly harmful
Neem extract	13.4 ± 6.40 ab	1. Innocuous	69.9 ± 8.14 abc	2. Slightly harmful
Paraffinic oil	11.0 ± 4.74 ab	1. Innocuous	69.6 ± 10.11 abc	2. Slightly harmful
Azoxystrobin	13.1 ± 5.32 ab	1. Innocuous	62.6 ± 10.11 abc	2. Slightly harmful
Berberine + Argemone	10.8 ± 3.65 ab	1. Innocuous	53.1 ± 10.33 abc	2. Slightly harmful
Thiophanate	18.4 ± 3.62 ab	1. Innocuous	44.3 ± 11.63 abc	2. Slightly harmful
Dispersant	1.46 ± 0.68 b	1. Innocuous	14.3 ± 1.92 bc	1. Innocuous
Water (Control)	0 ± 0 b	1. Innocuous	0 ± 0 c	1. Innocuous
χ^2^	33.20		59.59	
*p* value	0.0016		<0.0001	
df	13		13	

Values represent mean ± standard error (SE). Within each column, means followed by the same letter are not significantly different according to Dunn’s test (*p* > 0.05).

**Table 3:** Mean (±SE), percentage of parasitized host eggs that turned black, adult emergence percentage and development time (egg to adult) of *T. exiguum*, when parasitism occurred before the host eggs were treated with 10 pesticides.

Treatments	Parasitism Expression	Adult Emergence	Time to Emergence (Days)
Control (water)	96 ± 2.4 a	96 ± 2.4 a	11.0 ± 0.0
Metalaxyl	49.7 ± 2.1 b	49.7 ± 2.1 b	12.0 ± 0.7
Neem	82.33 ± 0.5 a	82.33 ± 7.5 a	11.4 ± 0.5
Acephate	43.3 ± 8.6 b	43.3 ± 8.6 b	10.0 ± 0.0
Hexythiazox	49.7 ± 5.6 b	44 ± 8.3 b	11.0 ± 0.0
Aceite parafínico	31.05 ± 6.6 b	31.05 ± 6.6 b	9.7 ± 0.6
Argemona + Berberina	40.89 ± 2.3 b	40.89 ± 2.3 b	11.2 ± 0.5
Abamectin	32.0 ± 4.6 b	26 ± 1.1 b	11.7 ± 0.6
λ-Cyhalothrin	22.0 ± 5.3 b	14.7 ± 8.4 b	11.3 ± 0.2
Thiabendazol	9.45 ± 5.9 bc	9.45 ± 5.9 b	12.0 ± 0.4
Potassium soap	0 ± 0.0 c	0 ± 0.0 c	—
*p* value	0.00178 **	0.000146 ***	0.087 ns

Values are mean ± SE. Means followed by the same letter are not significantly different (Scott–Knott test, *p* > 0.05); Codes: *** *p* < 0.001, ** *p* < 0.01, (trend) ns = not significant.

## Data Availability

Data will be made available on request.

## Supplementary Materials

The following supporting information can be downloaded at: https://www.mdpi.com/article/10.3390/insects17070728/s1.

## Use of Artificial Intelligence Tools in Scientific Writing

During the preparation of this work, Artificial intelligence tools were used in the preparation of this manuscript. Specifically, Claude Sonnet 4.6 (Anthropic, San Francisco, CA, USA) was employed for language editing of the document, while all scientific content, data interpretation, and conclusions remained solely the responsibility of the authors. Figures presented in this study were created using BioRender (BioRender.com, accessed on 26 March 2026), an AI-powered scientific illustration platform, based on original photographs taken by the authors during the experimental procedures.

## Abbreviations

The following abbreviations are used in this manuscript:

IPM	Integrated Pest Management
FDR	False Discovery Rate correction
SE	Standard error
IOBC	International Organisation for Biological Control
